# Allograft Versus Autograft in Anterior Cervical Discectomy and Fusion: A Propensity-Matched Analysis

**DOI:** 10.7759/cureus.22497

**Published:** 2022-02-22

**Authors:** Evelyn Ouro-Rodrigues, Anirudh K Gowd, Omar Ramos Williams, Peter B Derman, Siamak Yasmeh, Wayne K Cheng, Olumide Danisa, Joseph N Liu

**Affiliations:** 1 Orthopedic Surgery, Loma Linda University School of Medicine, Loma Linda, USA; 2 Orthopedic Surgery, Wake Forest Baptist Medical Center, Winston Salem, USA; 3 Orthopedic Surgery, Loma Linda University Medical Center, Loma Linda, USA; 4 Spine Surgery, Texas Back Institute, Plano, USA; 5 Orthopedics, Jerry L. Pettis VA Medical Center, Loma Linda, USA; 6 Orthopedic Spine, Loma Linda University Medical Center, Loma Linda, USA; 7 Orthopedic Surgery, University of Southern California Keck School of Medicine, Los Angeles, USA

**Keywords:** length of stay, complications, quality improvement, discectomy, autograft, allograft

## Abstract

Objective

To compare the 30-day complication rate associated with allograft versus autograft in anterior cervical discectomy and fusion (ACDF) and to determine preoperative factors that may influence complication rate.

Methods

The American College of Surgeons National Surgical Quality Improvement Program database was retrospectively queried from 2014 to 2017 for all procedures with CPT codes for ACDF (22551). Patients undergoing ACDF with either autograft or allograft were matched by propensity scores based on age, sex, body mass index, and comorbidities. The incidence of adverse events in the 30-day postoperative period was compared.

Results

A total of 21,588 patients met the inclusion and exclusion criteria. Following the 10:1 propensity match, 17,061 remained in the study (1,551 autograft and 15,510 allograft). The overall adverse event rate was 3.18%; 3.48% for autograft and 3.15% for allograft (P=0.494). Autograft had a significantly greater incidence of extended length of stay (>2 d) (LOS) (48.9% vs 34.8%; P<0.001). Multivariate analysis suggested that autograft selection was associated with extended LOS (OR 1.4; 95% CI 1.3-1.5).

Conclusion

The results of this study provide information regarding how graft selection can relate to extended hospital LOS and influence postoperative complications. Extended LOS may be associated with worse patient outcomes and increase the overall cost of care. Further study should be performed to determine which patients would benefit from autograft versus allograft with regards to long-term outcomes, in consideration of these increased short-term complications.

## Introduction

Anterior cervical discectomy and fusion (ACDF) is a common procedure to treat cervical radiculopathy and myelopathy [1-2]. As such, from 2006 to 2013, the annual number of ACDF surgeries increased by 5.7% [3]. Structural autograft was historically considered the “gold standard” for spinal fusion; however, the harvest of autogenous iliac crest bone graft involves a separate incision and has been associated with increased morbidity, operative time, hospital stay, and postoperative recovery [4-6].

Allograft may avoid complications associated with harvesting iliac crest autograft and has been used with increasing frequency in ACDF surgery [2]. Disadvantages associated with the use of allograft include increased risk of pseudarthrosis, presumably due to the absence of osteogenic factors that promote fusion as well as increased risk of infection or disease transmission and increased cost [7-9]. The rate of successful bone fusion has been reported to be 87-94.3% with allograft and 83-98% with autograft; however, the majority of postoperative clinical outcome evaluations found no statistical significance between graft type [6, 10, 11].

Given the ever-rising cost of healthcare, it is important to determine if the choice of graft type can significantly alter the overall cost of ACDF surgery. Further knowledge of peri- and postoperative complications of allograft versus autograft is required to determine the cost-effectiveness of graft choice [4]. The purpose of this study is to compare the early complication risk associated with allograft versus autograft in ACDF and determine which preoperative factors may influence complication rate.

## Materials and methods

The American College of Surgeons National Surgical Quality Improvement Program (ACS-NSQIP) database was retrospectively queried between 2014 and 2017 to provide the power for an appropriate analysis. The ACS-NSQIP is a prospectively maintained database of 30-day surgical complications following surgery. This database is formed from a growing network of 674 participating hospitals. The database assures quality by requiring participating hospitals to staff clinical reviewers.

The ACS-NSQIP was queried by patients using CPT codes for anterior cervical interbody fusion, with discectomy and decompression (22551). Prior to 2011 two codes (63075 for the discectomy and 22554 for the fusion) were used. After 2011, CPT combined these two procedures into one code, 22551, for the first level of fusion and discectomy and 22552 for subsequent levels.

The inclusion years were selected to limit bias from improper coding. Codes 60374 and 22554 are used in cases where only those individual procedures are performed, and they are not combined. For this study, codes 60375 and 22554 were excluded. The number of levels and interbody cages were queried separately using their respective CPT codes, 22552 and 22853.

Autograft was selected as the graft choice with either morselized autograft, structural autograft, using CPT codes 20937 and 20938, respectively. Allograft was selected as the graft choice with either morselized allograft, structural allograft, local autograft, or iliac crest bone marrow aspirate using CPT codes 20930, 20931, 20936, or 38220, respectively.

Matched groups of ACDF with autograft and allograft were established with the following variables: age, sex, body mass index (BMI), proportion of smokers, inpatient status, diabetics requiring therapy with insulin, dyspnea at moderate exertion, hypertension requiring medication, congestive heart failure within 30 days prior to surgery, steroids/immunosuppressants for a chronic condition, bleeding disorders, and disseminated cancer.

Demographic variables such as age, sex, BMI, operative time, dependent functional status, total length of stay, inpatient status, and comorbidities were reported for all included patients. The incidence of adverse events was reported as either mortality, wound dehiscence, sepsis, pulmonary embolism, myocardial infarction, anemia requiring transfusion, deep vein thrombosis (DVT), urinary tract infection (UTI), pneumonia, unplanned intubation, surgical site infection, extended length of stay (LOS), and return to the operating room. In agreement with prior studies, extended LOS was defined as ≥ 2 days [4, 12, 13].

Statistical analysis was performed using RStudio software version 1.3.959 (R Foundation for Statistical Computing, Vienna, Austria). A 10:1 propensity match was performed using the nearest-neighbor method of the MatchIt package of RStudio. This function creates propensity scores using composites of each variable included within the match and then equates these scores across both groups. Student’s t-test and Fisher’s exact test were performed to verify the appropriateness of the match for discrete and categorical variables, respectively. The incidence of complications for total adverse events, individual adverse events, extended length of stay, and reoperation rate were compared between autograft and allograft using Fisher’s exact test. Multivariate step-down Poisson regression with robust error variance was used to determine significant variables that are associated with complications following ACDF. Odds ratios with 95% confidence intervals were calculated. Overall significance was set to P < .05.

## Results

A total of 21,588 patients met the inclusion and exclusion criteria. There were 1,551 patients in the autograft group: 1,142 morselized autograft and 414 structural autograft. There were 20,037 patients in the allograft group: 9,904 structural allograft, 8,612 morselized allograft, 6,491 local autograft, and 753 iliac crest bone marrow aspirate. Prior to propensity match, patients receiving autograft and allograft differed with respect to body mass index, operative time, length of stay, inpatient status, comorbid diabetes mellitus, chronic obstructive pulmonary disease (COPD), and current smoker status. Following the 10:1 propensity match, 17,061 remained in the study (1,551 autograft and 15,510 allograft). The average age was 55.0±11.5 years. The average BMI was 29.9±6.4. Patients receiving autograft and allograft, respectively, differed with respect to operative time in minutes (140 ± 77.5 vs 130 ± 69.4; P<0.001) and total length of stay (2.51 ± 4.19 vs 2.06 ± 3.25; P<0.001; Table 1).

**Table 1:** Demographic and comorbidity characteristics for patients undergoing ACDF ACDF: Anterior Cervical Decompression and Fusion; COPD: Chronic obstructive pulmonary disease

	Autograft Unmatched	Rate %	Allograft Unmatched	Rate%	P-Value	Autograft Matched	Rate %	Allograft Matched	Rate %	P-Value
Demographics										
No. of patients	1551		20037			1551		15510		
Age, yr	55.0 ± 11.6		55.0 ± 11.4		0.952	55.0 ± 11.6		55.0 ± 11.5		0.982
BMI	30.0 ± 6.4		30.5 ± 6.7		0.001	30.0 ± 6.4		29.9 ± 6.4		0.774
Operative time, min	140 ± 77.5		126.6 ± 67.0		<0.001	140 ± 77.5		130 ± 69.4		<0.001
Female sex	773	49.84%	10107	50.44%	0.654	773	49.84%	7706	49.68%	0.915
Dependent functional status	1	0.06%	30	0.15%	0.433	1	0.06%	22	0.14%	0.717
Levels	1.65 ± 0.69		1.63 ± 0.67		<0.001	1.65 ± 0.69		1.65 ± 0.68		0.905
Total length of stay (days)	2.51 ± 4.19		1.97 ± 3.19		<0.001	2.51 ± 4.19		2.06 ± 3.25		<0.001
Inpatient	1279	82.46%	14260	71.17%	<0.001	1279	82.46%	12766	82.31%	0.917
Comorbidities										
Diabetes mellitus	72	4.64%	1258	6.28%	0.008	72	4.64%	644	4.15%	0.353
Dyspnea on exertion	66	4.26%	952	4.75%	0.419	66	4.26%	632	4.07%	0.737
Hypertension	719	46.36%	9413	46.98%	0.654	719	46.36%	7119	45.90%	0.749
Congestive Heart Failure	6	0.39%	50	0.25%	0.294	6	0.39%	47	0.30%	0.479
COPD	93	6.00%	873	4.36%	0.004	93	6.00%	785	5.06%	0.117
Current smoker	469	30.24%	5376	26.83%	0.004	469	30.24%	4510	29.08%	0.349
Steroid Use	53	3.42%	692	3.45%	1	53	3.42%	520	3.35%	0.882
Bleeding disorder	14	0.90%	258	1.29%	0.256	14	0.90%	121	0.78%	0.549
Metastatic cancer	0	0.00%	26	0.13%	0.256	0	0.00%	0	0.00%	1

The overall adverse event rate was 3.18%; 3.48% for autograft and 3.15% for allograft (P=0.494). The most common complication was extended LOS (n=6158, 36.1%). Autograft had a significantly greater incidence of extended LOS (48.9% vs 34.8%; P<0.001; Table 2).

**Table 2:** Incidence of adverse events DVT: Deep vein thrombosis; UTI: Urinary tract infection

	Autograft	Rate %	Allograft	Rate %	P-Value	Overall Rate %
Any adverse event	54	3.48%	489	3.15%	0.494	3.18%
Mortality	0	0.00%	0	0.00%		0.00%
Wound dehiscence	1	0.06%	7	0.05%	0.534	0.05%
Sepsis	9	0.58%	61	0.39%	0.292	0.41%
Pulmonary Embolism	4	0.26%	38	0.25%	0.790	0.25%
Myocardial infarction	1	0.06%	29	0.19%	0.518	0.18%
Transfusion	10	0.64%	53	0.34%	0.075	0.37%
DVT	8	0.52%	68	0.44%	0.687	0.45%
UTI	10	0.64%	84	0.54%	0.588	0.55%
Pneumonia	19	1.23%	130	0.84%	0.116	0.87%
Unplanned intubation	8	0.52%	118	0.76%	0.351	0.74%
Surgical site infection	13	0.84%	89	0.57%	0.223	0.60%
Return to operating room	31	2.00%	342	1.71%	0.364	2.00%
Extended length of stay (>2 d)	758	48.87%	5400	34.82%	<0.001	36.09%

In the multivariate logistic regression, the following factors were associated with any adverse event when controlling for all other variables: age >55 years, female sex, BMI>30, diabetes mellitus, COPD, hypertension, and bleeding disorder (Table 3).

**Table 3:** Multivariate analysis: risk factors for any adverse event COPD: Chronic obstructive pulmonary disease

	Odds Ratio	95% Confidence Interval	P-value
Allograft vs. Autograft	1.1	0.8-1.4	0.655
Age >55	1.6	1.3-1.9	<0.001
Female sex	0.8	0.7-1.0	0.017
BMI > 30	0.8	0.6-0.9	0.005
Diabetes mellitus	1.6	1.1-2.2	0.005
Dyspnea	1.1	0.7-1.5	0.778
Congestive Heart Failure	2.1	0.9-4.5	0.069
COPD	2.0	1.5-2.6	<0.001
Hypertension	1.4	1.2-1.7	<0.001
Dependent functional status	2.5	0.8-7.9	0.115
History of smoking	1.1	0.9-1.3	0.278
Metastatic cancer	NA	NA	NA
Bleeding disorder	2.1	1.2-3.6	0.01
Steroid Use	1.4	0.9-2.0	0.104

When accounting for all other covariates, autograft selection was associated with extended LOS (OR 1.4; 95% CI 1.3-1.5) (Table 4).

**Table 4:** Multivariate analysis: variables associated with extended length of stay COPD: Chronic obstructive pulmonary disease

	Odds Ratio	95% Confidence Interval	P-value
Allograft vs. Autograft	1.4	1.3-1.5	<0.001
Age >55	1.3	1.2-1.3	<0.001
Female sex	1.1	1.0-1.2	<0.001
BMI > 30	1.0	1.0-1.1	0.236
Diabetes	1.2	1.1-1.4	<0.001
Dyspnea	1.1	1.0-1.2	0.182
Congestive Heart Failure	1.3	1.0-1.9	0.087
COPD	1.1	1.0-1.2	0.067
Hypertension	1.1	1.0-1.2	<0.001
Dependent functional status	2.1	1.3-3.2	0.002
History of smoking	1	1.0-1.1	0.244
Metastatic cancer	NA	NA	NA
Bleeding disorder	1.4	1.1-1.7	0.004
Steroid Use	1.1	1.0-1.2	0.285

## Discussion

The principal findings of the study demonstrate that the overall incidence of any adverse event following ACDF in the post-operative 30-day period was relatively low at 3.01%, with no significant difference between groups that had allograft and autograft. However, after propensity matching and when accounting for all other covariates, autograft selection was associated with a greater incidence of extended LOS. The findings of the study demonstrate that ACDF with allograft has significant short-term advantages over ACDF with autograft.

It is not surprising that autograft was associated with a higher rate of extended length of stay. Prior research has demonstrated that approximately 50% of patients that undergo autograft bone harvest have ambulation difficulties and 26% have lasting chronic pain [14], which may require additional hospital days to safely prepare patients for discharge home with adequate pain control. This increased stasis may also explain the slight increase in the incidence of pulmonary embolism in patients treated with autograft. Furthermore, surgeons are more cautious about using pharmacologic anticoagulation due to possible postoperative hematoma that may result in acute postoperative airway complications and spinal cord compression.

Length of hospital stay (LOS) is one of the major variables that accounts for the difference in cost between patients undergoing ACDF surgery with up to 76% of total ACDF surgery cost attributed to post-surgical hospitalization [4]. Prior literature shows in-patient hospital charges, excluding surgical charges, for ACDF surgery ranged from $15,113 to $76,687, with most of the variance attributed to differences in LOS [15]. The initial cost of allograft can be up to $2,552 per patient [15], compared to iliac autograft which has no significant additional cost except for the added surgical time required for autograft harvest and preparation. However, multiple studies, including the present study, have now shown that autograft is associated with longer operative time, increased rate of complications, and extended LOS, all of which ultimately contribute to higher costs [6]. Cost-effectiveness analysis has demonstrated that allograft versus autograft offers a benefit in quality of life at a cost of 496 dollars per quality of life year (QALY) [16]. It is important to note, this study did not consider additional hospital resource use or complications associated with iliac autograft harvesting. If all complications, associated costs, and patient satisfaction were included in the model, it would likely decrease the cost per QALY of allograft with respect to autograft even further. The authors suggested that if autograft harvest morbidity could be better accounted for in their model, allograft might overtake autograft in cost-effectiveness analysis [16]. Other studies have shown that ACDF with autograft can cost up to double the amount of ACDF with allograft due to the increased costs associated with donor site morbidity [17]. Overall, the short-term advantages of ACDF with allograft must be paired with the higher cost of allograft and potential long-term complications when discussing graft selection with patients preoperatively.

In discussing graft choice, patient satisfaction must also be considered. One prior study examined patient satisfaction following ACDF surgery and found that based on pain and overall level of comfort, almost all patients favored allograft over autograft [18]. All patients enrolled in this study had previously undergone ACDF with autograft and were followed after revision surgery using a synthetic graft substitute, specifically tricalcium phosphate/hydroxyapatite (TCP/HA) composite within a polyetheretherketone (PEEK) cage [18]. Radiological studies concluded that all patients except one achieved post-operative fusion by nine months [18]. The implications of avoiding donor site morbidity and post-operative pain could drastically affect patient outcomes by decreasing the cost of opioids prescribed. Allograft may also pose an advantage for patients over autograft in decreasing ambulation difficulties, thereby lowering the cost of extended time off from work. Bundled payments seek to minimize the cost of healthcare by replacing the fee-for-service model with a fixed fee paid to hospitals for the surgical hospitalization and subsequent 90-day postoperative period [19]. This model provides an incentive for the surgeon to optimize patient care while minimizing extended LOS and postoperative complications. The findings of the present study suggest the use of allograft in ACDF may decrease LOS and therefore resource utilization, a potential strategy for economic sustainability.

This study has several limitations that should be addressed. The primary limitation of this study is that it is limited to the ACS-NSQIP database and complication rates are not reported past 30 days, such as the risk of pseudoarthrosis. Furthermore, the factors that influence the surgeon’s decision to use either autograft or allograft in each case are unavailable for study and may confound the results and introduce bias. Another potentially confounding feature in ACDF would be the use of cages, although the primary reported complication resulting from cages is dysphagia [20]. Since there is no ICD-10 code specifically for ACDF revision this study could not control for revision versus primary surgery. Even with these limitations, we believe that the present findings provide valuable information regarding the effect of graft selection on patient outcome and potential complications.

## Conclusions

The results of this study provide information regarding how graft selection can extend hospital LOS and influence postoperative complications. Extended LOS may be associated with worse patient outcomes and increase the overall cost of care. Further study should be performed to determine which patients would benefit from autograft versus allograft with regards to long-term fusion, in consideration of these increased short-term complications.
